# Characterization of Oilseed Lipids from “DHA-Producing *Camelina sativa*”: A New Transformed Land Plant Containing Long-Chain Omega-3 Oils

**DOI:** 10.3390/nu6020776

**Published:** 2014-02-21

**Authors:** Maged P. Mansour, Pushkar Shrestha, Srinivas Belide, James R. Petrie, Peter D. Nichols, Surinder P. Singh

**Affiliations:** 1 CSIRO Food Futures Flagship, Marine and Atmospheric Research, Castray Esplanade Hobart, Tasmania 7000, Australia; E-Mail: Peter.Nichols@csiro.au; 2 CSIRO Food Futures Flagship, Plant Industry, P.O. Box 1600, Canberra ACT 2601, Australia; E-Mails: Pushkar.Shrestha@csiro.au (P.S.); Srinivas.Belide@csiro.au (S.B.); James.Petrie@csiro.au (J.R.P.); Surinder.Singh@csiro.au (S.P.S.)

**Keywords:** DHA, docosahexaenoic acid, *Camelina sativa*, omega-3, long-chain PUFA, triacylglycerol, polar lipids, glycolipids, phospholipids, fish oils

## Abstract

New and sustainable sources of long-chain (LC, ≥C_20_) omega-3 oils containing DHA (docosahexaenoic acid, 22:6ω3) are required to meet increasing demands. The lipid content of the oilseed of a novel transgenic, DHA-producing land plant, *Camelina sativa*, containing microalgal genes able to produce LC omega-3 oils, contained 36% lipid by weight with triacylglycerols (TAG) as the major lipid class in hexane extracts (96% of total lipid). Subsequent chloroform-methanol (CM) extraction recovered further lipid (~50% polar lipid, comprising glycolipids and phospholipids) and residual TAG. The main phospholipid species were phosphatidyl choline and phosphatidyl ethanolamine. The % DHA was: 6.8% (of total fatty acids) in the TAG-rich hexane extract and 4.2% in the polar lipid-rich CM extract. The relative level of ALA (α-linolenic acid, 18:3ω3) in DHA-camelina seed was higher than the control. Major sterols in both DHA- and control camelina seeds were: sitosterol, campesterol, cholesterol, brassicasterol and isofucosterol. C_16_–C_22_ fatty alcohols, including iso-branched and odd-chain alcohols were present, including high levels of iso-17:0, 17:0 and 19:0. Other alcohols present were: 16:0, iso-18:0, 18:0 and 18:1 and the proportions varied between the hexane and CM extracts. These iso-branched odd-chain fatty alcohols, to our knowledge, have not been previously reported. These components may be derived from wax esters, or free fatty alcohols.

## 1. Introduction

There are many beneficial health effects in humans of omega-3 long-chain (≥C_20_) polyunsaturated fatty acids (omega-3 LC-PUFA, also termed LC omega-3 oils). The major bioactive LC omega-3 are EPA (eicosapentaenoic acid, 20:5ω3) and DHA docosahexaenoic acid, 22:6ω3). These are largely obtained through dietary consumption of seafood, primarily fish and fish oil nutraceuticals. The positive effects are across a range of degenerative and inflammatory disorders such as: heart disease, stroke, rheumatoid arthritis, asthma and some cancers, diabetes mellitus, multiple sclerosis, dementia and clinical depression [1,2,3]. LC omega-3 oils are also important in infant nutrition and are present in high concentrations in brain and retina and are important in the development, health and correct functioning of these organs [4,5]. They are also nutritionally important for the survival, growth and general health of aquaculture species particularly at the larval stage [6]. Highly purified or enriched LC omega-3 oils are also sought after for their bioactive function as potential pharmaceutical products [7].

Future supplies of fish oil derived LC omega-3 oils from fisheries is unlikely to be able to meet increasing demands for their inclusion in aquafeeds, foods, nutraceuticals, or for enrichment to highly purified EPA and DHA, as pharmaceutical bioactives and other products [8,9]. Hence, there is a need to develop new and sustainable sources to supplement oils extracted from wild fish harvests.

It has been recognized that marine fish do not have the capacity to synthesize these oils, rather it is microalgae, at the base of the marine food web, that have the ability to produce these oils [10,11]. Microalgae contain the genes for the various elongase and desaturase enzymes that are responsible for the synthesis of LC omega-3 oils. Members from the brown algal line, including for example the algal class dinoflagellates in particular, produce high proportions of DHA [12,13,14,15]. These health-benefitting oils are then accumulated up the food chain into various seafoods, including shellfish and finfish.

The search for new and sustainable sources of LC omega-3 oils has led researchers to attempt to transform land plants with microalgal LC omega-3 genes. As a result, a suite of microalgal genes have successfully been incorporated into a variety of land plants including tobacco leaf [16] and *Arabidopsis* seed [17,18] as a proof of concept, showing that transformed terrestrial plants can serve as an alternative supply of these oils. A little known oilseed plant, *Camelina sativa*, a member of the Cruciferae (Brassicaceae) family is an ancient plant native to Europe and Central Asian areas. It is cultivated as an oilseed crop and used in animal feed mainly in Europe and North America and is known as camelina, gold-of-pleasure or false flax. It has several unique and desirable features which give it a competitive advantage over other commercial oilseed crops such as canola, soybean, and sunflower [19]. It grows well in semiarid, marginal and saline soils and unlike commercial oilseed crops, does not have high nutrient requirements, can tolerate insects and weeds and survive winter sowing and frost and freeze-thaw cycles after emergence during late winter and spring. *Camelina sativa* seeds can produce up to 40% oil containing high proportions of α-linolenic acid (ALA, 28%) and linoleic acid (LA, 19%) [19], which makes this plant a good candidate for transformation into a LC omega-3 oil producing land plant. A new DHA-producing *Camelina sativa* oilseed plant transformed with a suite of microalgal LC omega-3 genes sourced from several target microalgal strains has been recently reported by Petrie *et al*. [20]. The synthesis of DHA occurs by the elongation and desaturation of C_18_ PUFA through EPA and not by retro-conversion of 24:6ω3. It was also determined by ^13^C NMR regiospecificity analysis of the TAG-containing oil that DHA is preferentially esterified at the *sn*-1,3 positions of the TAG molecule [20]. Here for the first time we present the lipid class, fatty acid, sterol and fatty alcohol composition of this new transformed DHA-producing *Camelina sativa* oilseed (hereafter termed DHA camelina) and carry out a descriptive analysis with reference to an unmodified *Camelina sativa* control seeds.

## 2. Experimental Section

### 2.1. Lipid Extraction

Details of the *Camelina sativa* seed, including: source, the full details of the gene construct inserted, which is composed of a series of elongase and desaturase genes, the metabolic pathway and methods used for the transformation to form the transgenic DHA-producing camelina oilseed event used in this study has been reported by Petrie *et al*. [20]. We used hexane as the extracting solvent since it is the industry standard and it preferentially extracts TAG-containing oil due to its solvating properties and very poor solubilization of polar lipids, particularly at room temperature. We did not use Soxhlet extraction so as to minimize any potential degradation of DHA due to heating for prolonged periods during reflux. No antioxidants were added during extraction or analysis. Subsequent extractions of the hexane-extracted crushed seed using chloroform-methanol were used to exhaustively recover residual TAG and un-extracted polar lipids to determine the effectiveness of extraction by hexane of the TAG-containing oil from the crushed seed. Transformed and control camelina seeds (130 g and 30 g, respectively) were wetted with hexane and crushed using an electric agate mortar and pestle (Retsch Muhle, Germany), transferred to a separatory funnel and extracted four times using a total of 800 mL hexane including an overnight third extraction. For each extraction, extracts were filtered to remove fines through a GFC glass fiber filter, and then rotary evaporated at 40 °C. The extracts were pooled and constituted the TAG-rich hexane extract.

Following extraction with hexane, the remaining meal was further extracted using chloroform-methanol (CM 1:1 v/v) as above, the meal was then removed by filtration and the combined extracts rotary evaporated. The pooled CM total crude extract was then dissolved using a modified Bligh and Dyer [21] one-phase methanol-chloroform-water mix (2:1:0.8 v/v/v). The phases were separated by the addition of chloroform-water (final solvent ratio, 1:1:0.9 v/v/v methanol-chloroform-water). The purified lipid was partitioned in the lower chloroform phase, concentrated using rotary evaporation and constituted the polar lipid-rich CM extract.

### 2.2. Lipid Class Analysis

Lipid classes of the hexane and CM extracts were analyzed by thin-layer chromatography with flame-ionization detection (TLC-FID; Iatroscan Mark V, Iatron Laboratories, Tokyo, Japan) [12] using hexane/diethyl ether/glacial acetic acid (70:10:0.1, v/v/v) as the developing solvent system in combination with Chromarod S-III silica on quartz rods and suitable calibration curves of representative standards obtained from Nu-Chek Prep, Inc. (Elysian, MN, USA). Data was processed using SIC-480II software (SISC Version: 7.0-E). Phospholipid species were separated by applying the purified phospholipid fraction (Section 2.3) obtained from silica column chromatography and developing the rods in chloroform/methanol/glacial acetic acid/water (85:17:5:2, v/v/v) prior to FID detection.

### 2.3. Separation of TAG, Glycolipid and Phospholipid Fractions from the CM Extracts

Silica gel 60 (100–200 mesh) (0.3–1 g) in a short glass column or Pasteur pipette plugged with glass wool was used to purify 10 mg of the purified CM lipid extract. The residual TAG fraction in the CM extract was eluted using 20 mL of 10% diethyl ether in hexane, the glycolipids eluted with 20 mL of acetone and the phospholipids eluted in two steps, first 10 mL of methanol then 10 mL of methanol-chloroform-water (5:3:2). This second elution was shown to increase the recovery of phospholipids [22]. The yield of each fraction was determined gravimetrically and the purity checked by TLC-FID. All extracts and fractions were stored in dichloromethane at −20 °C until further analysis by GC and GC-MS.

### 2.4. Fatty Acid Methyl Ester Preparation

Aliquots of the hexane and CM extracts were *trans*-methylated according to the method of Christie [23] to produce FA methyl esters (FAME) using methanol–chloroform–conc. hydrochloric acid (3 mL, 10:1:1, 80 °C, 2 h). FAME were extracted into hexane–chloroform (4:1, 3 × 1.8 mL). The meal (after hexane and CM extraction) was also *trans*-methylated and the value for total lipid was determined by adding the lipid contents of the hexane and CM extracts and the FAME content of the transmethylated meal after solvent extraction.

### 2.5. Sterol and Fatty Alcohol Derivatization

Samples (approximately 10 mg) from the TAG-rich hexane extract and the polar lipid-rich CM extract were saponified separately using 4 mL 5% KOH in 80% MeOH and heated for 2 h at 80 °C in a Teflon-lined screw-capped glass test tube. After the reaction mixture was cooled, 2 mL of Milli-Q water was added and the sterols and alcohols were extracted into 2 mL of hexane: dichloromethane (4:1, v/v, 3×) by shaking and vortexing. The mixture was centrifuged and the extract in the organic phase was washed with 2 mL of Milli-Q water by shaking and centrifugation. After taking off the top sterol containing organic layer the solvent was evaporated using a stream of nitrogen gas and the sterols and alcohols silylated using 200 µL of Bis(trimethylsilyl)trifluoroacetamide (BSTFA, Sigma-Aldrich) and heating for 2 h at 80 °C in a sealed GC vial; free hydroxyl groups were converted to their trimethylsilyl ethers.

### 2.6. GC and GC-MS Analysis

The sterol- and alcohol-OTMSi derivatives were dried under a stream of nitrogen gas on a heating block (40 °C) and re-dissolved in dichloromethane (DCM) immediately prior to GC/GC-MS analysis. The FAME and alcohol/sterol-OTMSi derivatives were analyzed by gas chromatography (GC) using an Agilent Technologies 6890A GC (Palo Alto, CA, USA) fitted with a Supelco Equity™-1 (Bellefont, PA, USA) fused silica capillary column (15 m × 0.1 mm i.d., 0.1 µm film thickness), an FID, a split/splitless injector and an Agilent Technologies 7683B Series auto sampler and injector. Helium was the carrier gas. Samples were injected in splitless mode at an oven temperature of 120 °C. After injection, the oven temperature was raised to 270 °C at 10 °C min^−1^ and finally to 300 °C at 5 °C min^−1^. Eluted compounds were quantified with Agilent Technologies ChemStation software (Palo Alto, CA, USA). GC results are subject to an error of ±5% of individual component area.

GC-mass spectrometric (GC-MS) analyses were performed on a Finnigan Trace ultra Quadrupole GC-MS (model: ThermoQuest Trace DSQ, Thermo Electron Corporation). Data was processed with ThermoQuest Xcalibur software (Austin, TX, USA). The GC was fitted with an on-column injector and a capillary HP-5 Ultra Agilient J & W column (50 m × 0.32 mm i.d., 0.17 µm film thickness, Agilent Technologies ( Santa Clara, CA, USA) of similar polarity to that described above. Individual components were identified using mass spectral data and by comparing retention time data with those obtained for authentic and laboratory standards. A full procedural blank analysis was performed concurrent to the sample batch.

## 3. Results

### 3.1. Total Lipid Content

The “DHA-producing camelina” seeds analyzed here contained slightly less total lipid (36% of seed wt.) than control wild-type *Camelina* (41% of seed wt.).

Of the total lipid, 31%–38% of lipid per seed weight was extracted by hexane for DHA- and control camelina, which accounted for 86% and 92% of total lipid, respectively (Table 1). The chloroform-methanol extraction post hexane extraction recovered a further 4.8% and 2.4% polar lipid-rich extract from the DHA- and control camelina, respectively and the residual lipid released by transmethylation of the remaining solvent extracted oilseed meal was 0.3% and 0.4% of seed weight, respectively.

**Table 1:** Lipid content (as % of seed weight) of DHA- and control *Camelina sativa* seeds after hexane extraction, post hexane chloroform-methanol (CM) extraction and subsequent transmethylation of the extracted meal.

Extract	DHA camelina	Control camelina
hexane	31.1	38.1
chloroform-methanol ^1^	4.8	2.4
residual lipid ^2^	0.3	0.4
Total lipid	36.2	40.9

^1^ Polar lipid rich extract containing glycolipid and phospholipid with some residual TAG obtained by CM extraction after hexane extraction of the meal; ^2^ Residual lipid (FAME) from transmethylated meal after hexane and CM extractions.

### 3.2. Lipid Class Analysis

The TAG-rich hexane extract (Section 2.1) contained 96% TAG in both DHA- and control *Camelina*. The post hexane chloroform-methanol extraction recovered residual TAG amounting to 44% and 13% (of the CM extract), respectively. The chloroform-methanol extracts were rich in polar lipids (glycolipids and phospholipids) amounting to 50% and 76% (of the CM extract) for the DHA- and control camelina, respectively (Table 2). The main phospholipid was phosphatidyl choline (PC) and accounted for 70%–79% of the total phospholipids followed by phosphatidyl ethanolamine (PE, 7%–13%) with smaller relative levels of phosphatidic acid (PA, 2%–5%) and phosphatidyl serine (PS, <2%). There were several other unidentified components separated in the phospholipid fraction (Table 3).

**Table 2:** Lipid class composition (% of total lipid obtained for each extraction step) of Hexane and CM extracts from DHA- and control *Camelina sativa* seeds.

Lipid class	DHA camelina		Control camelina	
	Hexane	Chloroform-methanol	Hexane	Chloroform-methanol
SE/WE/HC ^1^	1.0	1.4	1.0	1.4
TAG	95.6	44.2	96.0	13.1
FFA	0.9	1.3	0.8	1.4
UN ^2^	0.9	1.1	0.8	1.2
ST	0.5	0.7	0.4	0.4
MAG	0.7	1.1	0.8	6.2
PL	0.3	50.3	0.3	76.3
Total	100.0	100.0	100.0	100.0

Abbreviations: sterol esters (SE), wax esters (WE), hydrocarbons (HC), triacylglycerols (TAG), free fatty acids (FFA), unknown (UN), sterols (ST), monoacylglycerols (MAG), polar lipids (PL) consisting of glycolipids and phospholipids; ^1^ SE, WE and HC coelute with this system; ^2^ May contain fatty alcohols and diacylglycerols (DAG).

**Table 3:** Phospholipid composition (% of total phospholipids) of CM extracts from DHA- and control camelina seeds.

Phospholipid	DHA camelina	Control camelina
PA	2.1	4.7
UN 1	5.7	2.2
UN 2	-	1.1
UN 3	-	0.6
PE	13.2	6.8
PS	1.2	1.4
PC ^1^ + PI	69.5	78.9
UN 4	4.8	3.6
UN 5	3.4	1.6
Total	100.0	100.0

Abbreviations: Unknown (UN), phosphatidic acid (PA), phosphatidyl ethanolamine (PE), phosphatidyl serine (PS), phosphatidyl choline (PC), phosphatidyl inositol (PI); ^1^ PC is the major component and PI coelutes with PC.

**Table 4:** Fatty acid composition (% of total fatty acids) of various lipid extracts and fractions of DHA- and control *Camelina sativa*.

	DHA camelina							Control camelina				
	Hexane	Chloroform-methanol				Meal	Hexane	Chloroform-methanol				Meal
Fatty acid	TAG ^1^	Total ^2^	TAG ^3^	GL ^3^	PL ^3^	Residue ^4^	TAG ^1^	Total ^2^	TAG ^3^	GL ^3^	PL ^3^	Residue ^4^
16:1ω7	0.1	0.2	0.1	0.2	0.1	0.2	0.1	0.2	0.2	-	-	0.3
16:0	6.2	12.8	6.8	21.3	19.4	10.4	6.7	12.8	7.8	29.6	13.7	10.3
18:4ω3	3.7	3.3	3.4	2.1	2.9	3.6	-	-	-	-	-	-
18:2ω6	7.1	3.9	8.8	7.2	3.7	8.8	22.2	28.4	29.4	20.8	29.3	27.9
18:3ω3	41.9	50.3	39.9	38.6	54.1	38.9	32.0	20.6	19.7	13.0	12.3	20.0
18:1ω9	11.1	4.7	9.6	7.2	2.8	8.1	14.0	25.4	13.3	14.7	35.7	14.3
18:1ω7	1.4	2.3	2.1	3.7	3.4	2.8	1.0	1.5	2.2	4.0	2.8	2.2
18:0	3.2	4.0	3.0	4.5	5.7	3.1	3.0	2.7	2.9	5.7	3.6	2.7
20:5ω3	0.4	0.2	0.3	-	-	0.3	-	-	-	-	-	-
20:4ω3	0.4	0.4	0.4	-	0.2	0.3	-	-	-	-	-	-
20:2ω6	0.7	0.7	0.8	0.6	0.4	0.7	1.8	0.8	2.1	1.2	-	1.8
20:3ω3	0.8	1.2	0.9	0.6	1.3	0.5	0.9	0.3	-	-	-	0.4
20:1ω9/11	11.6	6.1	10.9	5.1	1.3	8.4	12.5	3.0	11.1	4.2	1.7	9.4
20:1ω7	0.6	0.8	1.4	0.6	0.2	1.1	0.6	0.6	2.6	1.3	-	1.8
20:0	1.3	0.8	1.4	0.6	0.1	1.4	1.5	0.7	2.3	1.4	-	1.8
22:6ω3	6.8	4.2	6.1	3.0	1.6	5.4	-	-	-	-	-	-
22:5ω3	0.3	1.1	0.4	0.6	1.4	0.3	-	-	-	-	-	-
22:1ω9	1.3	1.0	1.8	0.6	0.1	1.5	2.7	0.7	3.6	0.9	-	2.9
22:0	0.3	0.2	0.3	0.6	0.1	0.7	0.3	0.2	0.7	0.8	-	0.8
24:1ω9	0.3	0.4	0.4	0.6	0.3	0.6	0.3	0.6	0.7	0.9	0.5	1.0
24:0	0.1	0.4	0.2	0.9	0.4	1.1	0.1	0.4	0.5	1.4	0.4	1.3
Others ^5^	0.4	1.0	1.0	1.4	0.5	1.8	0.3	1.1	0.9	0.1	-	1.1
Total	100	100	100	100	100	100	100	100	100	100	100	100

Abbreviations: triacylglycerols (TAG), glycolipids (GL), phospholipids (PL); ^1^ TAG-rich hexane extract; ^2^ Total polar lipid-rich extract containing GL and PL from chloroform-methanol (CM) extraction post hexane extraction; ^3^ TAG, GL and PL from CM extraction separated by silica column chromatography; ^4^ Residual fatty acids released by transmethylation of camelina meal post hexane and CM extraction; ^5^ Sum of minor fatty acids.

### 3.3. Fatty Acid Composition

Generally we have found that total seed fatty acid composition obtained by direct transmethylation of whole seed is very similar to that of the TAG fraction, since the bulk of the lipids present in the seed occur in the form of TAG, hence total seed fatty acid composition is not reported here. In DHA camelina, the DHA was distributed in the major lipid fractions (TAG, phospholipids and glycolipids) and the proportion ranged between 1.6% and 6.8% with an inverse relationship between the proportions of DHA and ALA. The TAG-rich hexane extract of the DHA camelina contained 6.8% DHA and 41% ALA (Table 4). The polar lipid-rich chloroform-methanol extract contained 4.2% DHA and 50% ALA. Residual TAG from the polar lipid-rich chloroform-methanol extract contained 6% DHA and 40% ALA. The glycolipid fraction isolated from the chloroform-methanol extract contained 3% DHA and 39% ALA and the phospholipid fraction contained the lowest level of DHA (1.6%) and the highest levels of ALA (54%). The DHA camelina contained higher levels of ALA and lower levels of LA (linoleic acid, 18:2ω6) compared with the control camelina in the major lipid classes (TAG, glycolipids and phospholipids). The proportions of ALA and LA for DHA- and control camelina were: ALA 39%–54% and LA 4%–9% for DHA camelina and ALA 12%–32% and LA 20%–29% for control camelina. The relative level of eurucic acid (22:1ω9) was lower in all fractions in the DHA camelina than in the control (e.g., hexane extracts–1.3% *versus* 2.7%; Table 4).

### 3.4. Sterol Composition

The major sterols in both DHA- and control camelina were: 24-ethylcholesterol (sitosterol, 43%–54%), 24-methylcholesterol (campesterol, 20%–26%) with lower levels of cholesterol (5%–8%), brassicasterol (2%–7%), isofucosterol (∆5-avenasterol) (4%–6%), stigmasterol (0.5%–3%), cholest-7-en-3β-ol, (0.2%–0.5%), 24-methylcholestanol (campestanol, 0.4%–1%) and 24-dehydrocholesterol (0.5%–2%) (Table 5). These nine sterols accounted for 86%–95% of the total sterols, with the remaining components being unconfirmed sterols with partial identification obtained–including the number of carbons and double bonds.

**Table 5:** Sterol composition (% of total sterols) of DHA- and control camelina.

	DHA camelina		Control camelina	
Sterols	Hexane	CM ^1^	Hexane	CM ^1^
24-dehydrocholesterol	0.8	1.8	0.5	1.4
cholesterol	5.7	7.6	4.7	7.2
brassicasterol	4.4	6.5	1.9	4.2
cholest-7-en-3β-ol	0.2	0.5	0.3	0.4
campesterol	24.5	20.8	25.7	21.7
campestanol	0.4	1.1	0.4	0.9
stigmasterol	1.0	2.6	0.5	1.6
sitosterol	54.3	43.7	53.8	42.9
∆5-avenasterol (isofucosterol)	4.2	5.2	4.7	5.5
Sum	95.5	89.6	92.6	85.9
Others				
UN1 C28 1db	0.6	1.2	0.7	1.2
UN2 C29 1db	1.2	2.0	1.2	2.4
UN3 C29 2db	0.9	1.8	1.3	2.4
UN4 C28 1db	0.3	0.9	0.6	1.1
UN5 C30 2db	1.2	1.8	1.4	1.8
UN6 C29 1db + C30 2db	0.3	2.7	2.2	5.2
Sum	4.5	10.4	7.4	14.1
Total	100	100	100	100

Abbreviations: UN denotes unknown, C is number of carbon atoms and db denotes number of double bonds; ^1^ Polar lipid-rich extract containing glycolipids, phospholipids and residual TAG recovered by chloroform-methanol (CM) extraction post hexane extraction of crushed seed.

The overall sterol profiles were similar between DHA- and control camelina for both the hexane and chloroform-methanol extracts, although there were slightly higher levels of unknown sterols in the chloroform-methanol extracts of both the DHA- and control camelina than occurred in the hexane extracts (10%–14% and 4%–7%, respectively).

### 3.5. Fatty Alcohol Composition

A series of fatty alcohols from C_16_–C_22_, with accompanying iso-branched fatty alcohols, were identified in both the hexane and chloroform-methanol extracts (Table 6). Similar profiles were observed for the DHA- and control camelina, with some variation in the proportions of individual components observed. The odd-chain alcohols were present at higher levels in the chloroform-methanol extract (37%–38%) than in the hexane extract (16%–23%). Iso-17:0 (16%–38%) predominated over 17:0 (0.3%–5.7%). Another odd-chain alcohol present was 19:0 (4.5%–6.5%). Phytol, derived from chlorophyll, was the major aliphatic alcohol and accounted for 47% and 37% of the total fatty alcohols in the hexane fractions in the DHA- and control camelina, respectively. There were lower levels in the chloroform-methanol extract (9% and 12%, for DHA- and control camelina, respectively). Other alcohols detected included iso-16:0, 16:0, iso-18:0, 18:1, 18:0, with minor levels of iso-20:0, 20:1, 20:0, iso-22:0, 22:1 and 22:0 also present.

**Table 6:** Fatty alcohol composition (% of total sterols) of DHA- and control camelina.

Fatty alcohols	DHA camelina		Control camelina	
	Hexane	CM ^1^	Hexane	CM ^1^
*iso*-16:0	0.8	1.9	1.5	1.7
16:0	5.7	13.8	6.1	12.2
*iso*-17:0	16.4	37.1	23.5	38.6
17:0	3.8	0.3	5.7	1.0
*iso*-18:0	6.6	11.8	8.3	13.5
18:1	2.8	8.0	2.5	5.1
18:0	9.3	7.9	7.4	6.4
Phytol	47.1	9.1	37.4	11.7
19:0	4.5	5.6	5.1	6.7
*iso*-20:0	0.0	0.2	0.0	0.2
20:1	1.0	0.9	0.5	0.6
20:0	1.3	1.7	1.2	1.1
*iso*-22:0	0.0	0.2	0.0	0.2
22:1	0.0	0.5	0.0	0.3
22:0	0.8	1.0	0.7	0.5
Sum	100	100	100	100

^1^ Polar lipid-rich extract containing glycolipids and phospholipids with residual TAG obtained by chloroform-methanol (CM) extraction post hexane extraction of crushed seed.

## 4. Discussion

The oil content from camelina seeds can range from 25% to 48% [19,24,25,26] and can be dependent on the location where the plant is grown and the environmental conditions during growth. Previous researchers have published the fatty acid composition of camelina oil which is similar to what we report here for the control camelina [27]. Since this was only a descriptive analysis with no replication performed, we cannot yet conclusively determine whether the insertion of the omega-3 LC-PUFA microalgal-derived genes affected the oil yield. Hence, further work including replication would need to be done to determine if there is any statistical change in the oil yield. The results for the oil extraction suggest that slow crushing using a motorized mortar and pestle with multiple extractions with hexane at room temperature is effective in recovering the majority of the TAG oil.

In addition to the oil containing moderate levels of DHA, the DHA-containing camelina also had markedly higher levels of ALA in the major lipid classes (triacylglycerols, glycolipids and phospholipids) compared with the control camelina. This finding shows that the activity of the ∆-15 desaturase gene is considerably enhanced in DHA camelina [18,20] hence there is scope to increase the DHA content further by optimizing the elongation and desaturation of ALA. Variations in the level of ALA and DHA in the transformed plants may also be influenced by the effects of cultivar variety and other factors such as the quality of soil and climatic and weather conditions. It has also been reported that, in oilseed crops, the level of PUFA in general is promoted by low temperatures (winter and spring season) during the seed filling period, while at higher temperatures (summer season) the concentration of saturated fatty acids is higher [26].

Interestingly, there were some slight differences in the fatty acid profile and proportion of DHA in the various extracts and fractions with the DHA levels being higher in the TAG-rich hexane extract and TAG from CM extraction (6%–6.8%) and lower in the polar lipid fractions (3% in glycolipids and 1.6% in phospholipids), and 16:0 being higher in the polar lipid fractions of glycolipids and phospholipids from CM extraction (19%–21%) compared with the TAG-rich hexane extract and TAG from CM extraction (6%–7%). It is not known if the low levels of residual lipid present in the meal after solvent extraction (using hexane then chloroform-methanol) and recovered by transmethylation is derived from free solvent extractable lipid or bound lipid which is liberated only by hydrolysis under the hot acid methanolic transmethylation conditions. Future research will investigate this aspect.

The sterol composition of the camelina DHA and control camelina samples analysed here were similar to that found in refined camelina oil [28] with the same major sterols present, indicating that the added genes did not affect sterol synthesis. Previous workers reported the major sterols as being: cholesterol (4%), brassicasterol (3%), campesterol (21%), stigmasterol (2%), sitosterols (45%), ∆5-avenosterol (9%), cycloartenol (12%) and 24-methylene cycloartenol (3%). They also noted ten minor components which were unidentified due to their very low levels. We also observed several unidentified components at low relative levels (Table 5). Schwartz *et al*. [29] reported campestanol (1.6 mg), sitostanol (2.5 mg), stigmasta-5,24-dienol (6.2 mg), gramisterol + α-amyrin (1.9 mg), ∆7-avenosterol (trace levels) and citrostadienol (1.30 mg) in camelina oil in addition to the sterols identified by Shukla *et al*. [28]. They reported the levels of the common sterols (mg/100 g oil) in camelina oil as cholesterol (35 mg), brassicasterol (27 mg), campesterol (117 mg), stigmasterol (5.6 mg), sitosterol (300 mg), ∆5-avenosterol (37 mg), cycloartenol (10 mg) and 24-methylene cycloartenol (1.0 mg). The level of cholesterol in camelina oil was higher than occurs in most vegetable oils and brassicasterol is a characteristic sterol found in the Brassicaceae family of which camelina is one. 

Based on the combined analyses of a wide suite of lipid classes in DHA camelina and control camelina, it would seem then that the omega-3 LC-PUFA genes have, as expected, had little or no effect on the sterol composition. Further work will need to be carried out to determine the contributions of these sterols from the sterol ester and free sterol fractions, in each of the extracts, by separating the sterol ester and free sterol lipid classes and analysing them separately.

In relation to the presence of fatty alcohols, those with chain length C_16_–C_24_ have previously been observed in camelina after release from wax esters following saponification [30], but to our knowledge the presence of iso-odd-chain fatty alcohols such as found here, in both DHA camelina and control camelina, have not been reported previously. Further research needs to be performed to determine which fractions these alcohols are present in, e.g., in free or in an esterifiedform such as in a wax ester.

## 5. Conclusions

This is the first detailed report of the lipid composition of a new transformed terrestrial oilseed capable of producing DHA-containing (6.8% of total fatty acids) TAG oil with a simple fatty acid profile and a high preference of ω3 over ω6 LC-PUFA. Fish oils also have a high preference of ω3 over ω6 LC-PUFA. However, several key distinguishing features found in the transformed camelina fatty acid profile is the high level of α-linolenic acid (ALA, 18:3ω3) which is only a minor fatty acid in fish oils and the much lower levels of EPA (eicosapentaenoic acid, 20:5ω3) making the fatty acid profile of the oil unique. The chloroform-methanol extract is rich in polar lipids (glycolipids and phospholipid), since these are not extracted by hexane. Hence, the hexane extracted seed meal, from which most of the TAG was removed may be useful as an animal feed supplement (e.g., in aquaculture) or as a source of high value DHA-containing polar lipids (e.g., phosphatidyl choline containing DHA). The profiles of other lipid classes such as sterols and fatty alcohols were very similar to the control camelina. These results hold promise for the development and commercial production of new and sustainable terrestrial sources of LC omega-3 oils, which will supplement or in part replace LC omega-3 containing marine oils, hence alleviating pressure on wild harvest fisheries arising from increasing demand for these oils. Future research will be extended to canola and include similar detailed lipid class characterisation, examination of extraction efficiencies, oil stability and enrichment of DHA.
